# Effectiveness of the 23-valent polysaccharide pneumococcal vaccine against invasive pneumococcal disease in people 60 years or older

**DOI:** 10.1186/1471-2334-10-73

**Published:** 2010-03-18

**Authors:** Angel Vila-Corcoles, Olga Ochoa-Gondar, Jorge A Guzmán, Teresa Rodriguez-Blanco, Elisabet Salsench, Cruz M Fuentes, EPIVAC Study Group

**Affiliations:** 1 Primary Care Service of Tarragona-Valls. Institut Catalá de la Salut. Tarragona, Spain; 2 Department of Epidemiology and Biostatistics, IDIAP Jordi Gol Foundation, Barcelona, Spain; 3 Department of Medicine and Surgery, Faculty of Medicine and Health Sciences, Rovira i Virgili University, Tarragona, Spain

## Abstract

**Background:**

The 23-valent polysaccharide pneumococcal vaccine (PPV) is currently recommended in elderly and high-risk adults. However, its efficacy in preventing pneumococcal infections remains controversial. This study assessed the clinical effectiveness of vaccination against invasive pneumococcal disease (IPD) among people over 60 years.

**Methods:**

Population-based case-control study that included 88 case patients over 60 years-old with a laboratory-confirmed IPD (bacteraemic pneumonia, meningitis or sepsis) and 176 outpatient control subjects who were matched by primary care centre, age, sex and risk stratum. Adjusted odds ratios (ORs) for vaccination were calculated using conditional logistic regression, controlling for underlying conditions. Vaccine effectiveness was estimated as (1 - OR) ×100.

**Results:**

Pneumococcal vaccination rate was significantly lower in cases than in control subjects (38.6% *vs *59.1%; p = 0.002). The adjusted vaccine effectiveness was 72% (OR: 0.28; 95% CI: 0.15-0.54) against all IPD and 77% (OR: 0.23; 95% CI: 0.08-0.60) against vaccine-type IPD. Vaccination was significantly effective against all IPD in both age groups: 60-79 years-old (OR 0.32; 95% CI: 0.14-0.74) and people 80 years or older (OR: 0.29; 95% CI: 0.09-0.91). Vaccination appears significantly effective as for high-risk immunocompetent subjects (OR: 0.29; 95% CI: 0.11-0.79) as well as for immunocompromised subjects (OR: 0.12; 95% CI: 0.03-0.53).

**Conclusion:**

These findings confirm the effectiveness of the 23-valent PPV against IPD, and they also support the benefit of vaccination in preventing invasive infections among high-risk and older people.

## Background

*Streptococcus pneumoniae *is an important cause of morbidity and mortality worldwide, particularly in young children and elderly individuals, who are more susceptible to infection. The main reservoir of the microorganisms is the nasopharynx, and pneumococcal disease presentation depends on whether the bacteria spread to adjacent mucosal tissues causing mucosal infections (otitis, sinusitis, bronchitis and nonbacteremic pneumonias) or whether it invades the bloodstream, or other sterile sites, resulting in invasive pneumococcal disease (IPD), principally bacteraemic pneumonias, meningitis and sepsis[1].

For many years, control of pneumococcal infections was largely focused on case identification and antimicrobial treatment. Although these actions reduce the consequences of disease, they have little impact on the overall disease burden and have contributed to the increasing rates of antimicrobial resistance among *S. pneumoniae *strains. The need for preventive strategies with a broad public health impact has been recognized for several decades and the aim of finding an effective anti-pneumococcal vaccine has driven multiple efforts during this time[2,3].

The existence of more than 90 distinct serotypes (differing in their chemical compositions, potential immunogenicity and epidemiological impact on different population groups) has largely complicated the development and evaluation of anti-pneumococcal vaccines[4]. To date, only a 23-valent polysaccharide pneumococcal vaccine (PPV) for use in adults and a 7-valent conjugate pneumococcal vaccine (CPV) for use in infants are available in clinical practice, although two new CPVs (including 10 and 13 serotypes) will also be available shortly.

The currently available 23-valent PPV was licensed in 1983 and it is usually recommended for use in high-risk adults and elderly people. The vaccine contains capsular polysaccharide antigens from the 23 most dominant serotypes among clinical isolates of *S*. *pneumoniae*, accounting for approximately 90% of overall invasive infections in the adult population[5,6].

If we consider IPD, the most specific and severe manifestation of the pneumococcal infection, its incidence is low (approximately 10-20 cases per 100,000 all-age inhabitants)[1] and prospective randomized controlled trials (RCTs) generally failed to demonstrate a significant protective effect of PPV against this outcome. In contrast, several case-control and indirect cohort studies have found considerable effectiveness against IPD or pneumococcal bacteraemia. Several meta-analyses have produced varied results depending on which trials were included, but have generally concluded that PPV would be considerably effective in preventing IPD among immunocompetent adults [7-16]. In the last published meta-analysis, which includes only RCTs, Huss et al [17] have reported that trial quality explains a substantial proportion of the heterogeneity in the results of meta-analyses. They conclude that there is no evidence of vaccine protection in trials of higher methodological quality and suggest that PPV could be not efficacious against either IPD or pneumonia[17].

In Catalonia, a region in the northeast of Spain with a population of six million people, a publicly funded anti-pneumococcal vaccination program for high-risk adults and people over 65 years began in October 1999. Further, the vaccination program was extended for all individuals 60 years or older (with or without high-risk conditions) in 2002. Since then, a free 23-valent PPV is offered when the patients come to the Primary Health Care Centers (PHCCs) during the annual influenza vaccination campaigns or in any other visit throughout the year[18]. In order to evaluate the effectiveness of the antipneumococcal vaccination program, we performed a population-based case-control study among people over 50 years in our study area. The results of vaccine effectiveness against pneumonia (with or without bacteraemia) have been published[19]. In the present study we have evaluated the clinical effectiveness of the 23-valent PPV against IPD (bacteremic pneumonia, meningitis or sepsis) in the population subgroup 60 years or older, and according to the age and the risk strata of the patients.

## Methods

### Design, setting and study population

Population-based case-control study that included 88 patients aged 60 years or more with laboratory-confirmed invasive pneumococcal disease and 176 control subjects. The study was carried out in three districts (Tarragones, Alt Camp, Baix Penedes) belonging to the Health Region of Tarragona (a mixed residential-industrial region in the Mediterranean coast of Catalonia, Spain).

Case patients were identified from an active surveillance made in the participating PHCCs and Laboratory Departments of 3 reference hospitals in the study area (Joan XXIII, Santa Tecla and Pius Hospitals) from January 2002 to April 2007. The study was approved by the ethical committee of the Catalonian Health Institute (ID: FIS PS-050231) and was conducted in accordance with the general principles for observational studies.

### Data sources

The hospital discharge diagnoses databases, coded according to the International Classification of Diseases, 9th Revision, Clinical modification (ICD-9), were initially used to identify possible case patients. The institutional medical databases of each PHCC (which contain administrative data together with registries of medication prescriptions, medical conditions and chronic diseases associated with ICD-9 codes) were used to select potential control subjects in each participating PHCC. Primary care clinical records were used to investigate the vaccination status of case and control subjects, and they were also used to identify the presence of underlying conditions registered before the date of matching.

### Case patients

A case was defined as a patient aged 60 years or older, living in the study area, who had a laboratory-confirmed episode of IPD during the study period. IPD was defined as a patient from whom *S. pneumoniae *was obtained by culture of blood samples, CSF samples, or other normally sterile sites. IPD cases were identified on the basis of ICD-9 discharge codes for bacteremia (038.0, 038.2, 041.0, 041.2), meningitis (320.1) or pneumonia (481). Laboratory records were also used for identified cases of pneumococcal infections not detected in ICD-9 discharge codes.

All the cases were validated by review of the medical record with the use of standardized data-collection instrument. IPD case was considered if, on conclusion of the medical record review, the physician reviewer verified this diagnosis and it was not a readmission or another diagnosis.

### Control subjects

For each case patient, two control subjects were selected from the participating PHCCs. Control subjects were matched to their respective case patient by PHCC, age (birthday within +/- 3 years of the case patient's birthday), sex, and main chronic medical condition (using risk stratum which are defined below).

To identify appropriate control subjects, we performed searches of the computerized medical record system at each PHCC to obtain lists of potential control patients according to ICD-9 codes for every underlying condition, and then we randomly selected 2 control subjects from the list of potential control patients with the appropriate conditions. If the list of potential control subjects for a case patient had less than 2 persons, then we systematically expanded the criteria (firstly, for age until +/- 10 years; secondly, searching for adequate control subjects in the nearest PHCC.

### Risk strata

To ensure that matching was adequate, underlying medical conditions of the case patients were grouped into 3 risk strata on the basis of the degree of immunocompromise and risk for pneumococcal disease. Stratum 1 included persons with conditions associated with possible immunocompromise: immunodeficiency (including AIDS), asplenia, cancer (solid organ or haematological neoplasia), chronic nephropathy (nephrotic syndrome, renal failure, dialysis or transplantation), and long-term corticosteroid therapy (20 mg/day of prednisone or equivalent). Stratum 2 included patients without a level 1 condition but who had a history of chronic lung disease (chronic bronchitis, emphysema or asthma), liver disease (cirrhosis or alcoholic hepatitis), heart disease (congestive heart failure or chronic angina) and diabetes mellitus. Stratum 3 included patients without level 1 or level 2 conditions.

When a case was categorized as risk stratum 1, it was matched with two control patients who had disease or condition in risk stratum 1. When a case patient had one underlying condition and was categorized as risk stratum 2, control subjects were selected with the same medical condition. If a case patient had multiple underlying conditions, control patients were matched according to the first-listed condition above. If the case patient was risk stratum 3, then the control subjects were only matched according to their smoking or non smoking status.

### Vaccination history

Vaccination status of case and control subjects were determined by a review of the PHCCs' clinical records, which contain specially designated fields for pneumococcal and influenza vaccinations. We assumed that information in clinical records was complete, so a subject was considered as unvaccinated when a vaccination was not recorded.

Patients and their respective matched control subjects were considered as vaccinated against pneumococcus if the 23-valent PPV had been administered at least 14 days before the onset of the illness for the case patient. Similarly, influenza vaccination status was determined according to whether the subjects had received the flu vaccine in the autumn prior to the IPD date. The investigators who performed microbiological procedures and those who selected control subjects were unaware of the vaccination histories of the subjects.

### Case-control analysis

The differences between case and control groups were evaluated with the chi-squared or Students' test as appropriate. The crude association between the outcomes and vaccination was evaluated using the Mantel-Haenszel matched odds ratio (OR). Conditional logistic regression was used to calculate the adjusted matched ORs. In the initial regression models, we adjusted for: age, sex, lung disease, heart disease, stroke, diabetes, liver disease, renal disease, cancer, corticosteroid therapy, alcoholism, smoking and influenza vaccine status. To avoid potential residual confusion due to the use of age as a categorical variable, all the models have been adjusted for age as continuous.

We performed stratified analysis in cases occurred within influenza period (January-April) or non-influenza period (May-December), in two age groups (60-79 yrs, 80 yrs or more) and in each of the three risk strata. We checked for confounding factors, interactions and colinearity among the independent variables. All the models have been compared through the partial likelihood ratio test or the Akaike's information criterion (AIC). We evaluated the assessment of fit in the final models[20]. Vaccine effectiveness was calculated by (1-matched OR) ×100%. Statistical significance was set at p < 0.05 (two-tailed). The analyses were performed using Stata/SE Version 9.1. (Stata Corp.)

## Results

### Characteristics of the study population

Among the total 88 cases, eight (9.1%) were meningitis, four (4.5%) were bacteraemia with no apparent focus and 76 (86.4%) were bacteraemic pneumococcal pneumonias. Five cases of bacteremic pneumonia were managed as outpatient whereas the remaining 83 case patients were hospitalised (15 of which required admission to the intensive care unit). Overall case-fatality rate reached 18.2% (16/88).

Of the 53 identified serotypes, 44 (83%) were vaccine types, 4 (7.6%) were vaccine-related serotypes and 5 (9.4%) were non-vaccine serotypes. Of the remaining 35 isolates, 9 were not typable and 26 were not typed. Table 1 shows absolute numbers and proportions of the different identified serotypes.

**Table 1:** Absolute numbers and proportions of the different pneumococcal serotypes, according to they were included or not in the 23-valent polysaccharide pneumococcal vaccine (PPV) identified among case patients (n = 53).

S. pneumonia serotype	N (%)
**Serotypes included in 23-valent PPV**	
1	6 (11.3)
3	7 (13.2)
4	3 (5.6)
5	2 (3.8)
6B	3 (5.6)
7F	3 (5.6)
8	4 (7.5)
9V	2 (3.8)
12F	1 (1.9)
14	8 (15.1)
15B	1 (1.9)
19A	2 (3.8)
19F	2 (3.8)

**Vaccine-related serotypes**	
6A	2 (3.8)
23A	2 (3.8)

**Non-vaccine serotypes**	
13	1 (1.9)
16	1 (1.9)
31	2 (3.8)
38	1 (1.9)

Mean age of case patients was 73.2 years (SD: 10.8) and age of case patients ranged between 60-92 years. Overall, 54 cases (61.4%) were male and 37 (42.1%) were 80 years or older. Twenty-nine cases (32.9%) were assigned to risk stratum 1, 41 (46.6%) to risk stratum 2, and 18 (20.5%) to risk stratum 3.

The characteristics of the cases and control subjects are summarized in table 2. The pneumococcal vaccination rate was significantly lower in cases than in control subjects (38.6% *vs *59.1%; p = 0.002). As for the other characteristics the two groups were essentially similar, except for a slightly more significant prevalence of stroke among case patients than in control subjects (11.4% *vs *5.1%, p = 0.064) and a lower influenza vaccination rate in case patients than in control subjects (48.3% vs 64.8%, p = 0.010).

**Table 2:** Characteristics of subjects by case-control status (88 matched sets) to evaluate the clinical effectiveness of the 23-valent polysaccharide pneumococcal vaccine in preventing Invasive pneumococcal disease among Spanish people 60 years or older.

Characteristic	Cases (n = 88)	Controls^a^ (n = 176)	P-value^b^
**Age (yrs), mean (SD)**	73.2 (10.8)	72.8 (10.2)	0.612

**Sex male**	54 (61.4)	108 (61.4)	1.000

**Cancer**	12 (13.6)	32 (18.2)	0.350

**Chronic renal disease**	15 (17.0)	25 (14.2)	0.865

**Corticosteroid therapy**	4 (4.5)	10 (5.7)	0.698

**Chronic lung disease**	19 (21.6)	44 (25.0)	0.540

**Chronic liver disease**	7 (8.0)	16 (9.1)	0.758

**Chronic heart disease**	31 (35.2)	46 (26.1)	0.126

**Diabetes mellitus**	21 (23.9)	51 (29)	0.379

**Stroke**	10 (11.4)	9 (5.1)	0.064

**Alcoholism**	5 (5.7)	8 (4.3)	0.687

**Smoking**	18 (20.5)	27 (15.3)	0.298

**History of pneumococcal vaccination**	34 (38.6)	104 (59.1)	0.002

**History of influenza vaccination in autumn prior to the event**	42 (48.3)	114 (64.8)	0.010

NOTE: Data are numbers (percentage) of subjects, unless otherwise indicated. SD, standard deviation.

^a^Criteria for matching control subjects with case patients were extended in 9 sets for age (4-5 years in 3 sets, 6-7 years in 2 sets, and 8-10 years in 4 sets), and it was extended for nearest municipality or Primary Health Care Center in 14 sets.

^b ^P-values were calculated with the chi-squared test and Student's test, as appropriate.

### Effectiveness of the vaccination

In the crude analysis, vaccine effectiveness was 66% (95% CI: 37-82) against all IPD cases and it was 72% (95% CI: 33-89) against infections due to vaccine-types. In the multivariable analysis, the values of different results on the adjusted vaccine effectiveness were very similar to those observed in the crude analysis, observing that the adjusted vaccine effectiveness was 72% (95% CI: 46-85) against all IPD and 77% (95% CI: 40-92) against vaccine-type IPD. Table 3 shows vaccination histories in cases and control subjects, unadjusted and adjusted Odds Ratios, and estimates of effectiveness of the vaccination against vaccine-type IPD and all IPD.

**Table 3:** Vaccination histories in cases and control subjects, unadjusted and adjusted Odds Ratios, and estimates of effectiveness of pneumococcal vaccination against overall invasive pneumococcal disease (IPD) and IPD by vaccine types.

			Unadjusted analysis		Adjusted analysis	
**Type of infection**	**No. of subjects**	**No. (%) of vaccinated**	**OR^a ^(95% CI)**	**Effectiveness of vaccination % (95% CI)**	**OR^b ^(95% CI)**	**Effectiveness of vaccination % (95% CI)**

**IPD by vaccine types**					^(c)^	
Case patients	48	15 (31.3)	0.28 (0.11-0.67)	72% (33-89)	0.23 (0.08-0.60)	77% (40-92)
Control subjects	96	52 (54.2)	p = 0.005		p = 0.003	

**Overall IPD**					^(d)^	
Case patients	88	34 (38.6)	0.34 (0.18-0.63)	66% (37-82)	0.28 (0.15-0.54)	72% (46-85)
Control subjects	176	104 (59.1)	p = 0.001		p < 0.001	

NOTE: OR, odds ratio of vaccination versus no vaccination in cases and controls; Vaccine effectiveness = (1 - matched OR for vaccination)*100%; CI, confidence interval.

^a^Mantel-Haenszel matched Odds Ratio.

^b^Matched, adjusted Odds Ratio by conditional logistic regression model.

^c^Adjusted for age, smoking and stroke.

^d^Adjusted for age, chronic heart disease, alcoholism and smoking.

During the study period, 50 cases occurred within influenza period and 38 cases occurred within noninfluenza period. Within influenza period, the adjusted vaccine effectiveness was 73% (95% CI: 15-92) against all IPD, whereas it was 63% (95% CI:8-85) within noninfluenza period. Table 4 shows vaccination histories in cases and control subjects, unadjusted and adjusted Odds Ratios, and estimates of effectiveness of the vaccination against all IPD according to influenza and noninfluenza periods.

**Table 4:** Vaccination histories in cases and control subjects, unadjusted and adjusted Odds Ratios, and estimates of effectiveness of pneumococcal vaccination against invasive pneumococcal disease according to influenza and noninfluenza periods.

			Unadjusted analysis		Adjusted analysis	
**Type of infection**	**No. of subjects**	**No. (%) of vaccinated**	**OR^a ^(95% CI)**	**Effectiveness of vaccination % (95% CI)**	**OR^b ^(95% CI)**	**Effectiveness of vaccination % (95% CI)**

**Within influenza period (January-April)**					^(c)^	
Case patients	50	17 (34.0)	0.30 (0.12-0.73)	70% (27-88)	0.27 (0.08-0.85)	73% (15-92)
Control subjects	100	55 (55.0)	p = 0.008		p = 0.025	

**Within non-influenza period (May-December)**					^(d)^	
Case patients	38	17 (44.7)	0.38 (0.16-0.92)	62% (8-84)	0.37 (0.15-0.92)	63% (8-85)
Control subjects	76	49 (64.5)	p = 0.032		p = 0.033	

NOTE: OR, odds ratio of vaccination versus no vaccination in cases and controls; CI, confidence interval.

^a^Mantel-Haenszel matched Odds Ratio.

^b^Matched, adjusted Odds Ratio by conditional logistic regression model.

^c ^Adjusted for age, chronic heart disease and influenza vaccine in the prior Autumn.

^d ^Adjusted for age and diabetes mellitus.

In stratified analysis according to age subgroups, vaccination appears significantly effective against all IPD in both age groups 60-79 years (adjusted OR: 0.32; 95% CI: 0.14-0.74) and 80 years or older (adjusted OR: 0.29; 95% CI 0.09-0.91).

According to the risk strata of the patients, the reception of the 23-valent PPV was associated with a lower risk of all IPD for vaccinated subjects in the three risk strata, but vaccination effectiveness only reached statistical significance in risk stratum 1 (adjusted OR: 0.12; 95% CI: 0.03-0.53) and risk stratum 2 (adjusted OR: 0.29; 95% CI: 0.11-0.79).

Table 5 shows vaccination histories in case and control subjects, unadjusted and adjusted analysis of vaccination effectiveness against IPD according to age and risk strata of the patients.

**Table 5:** Unadjusted and adjusted analyses of vaccination effectiveness against IPD according to age and risk strata of the patients.

Characteristics of case patients	No. of sets	No. (% vaccinated)		Unadjusted analysis		Adjusted analysis	
		
		Case patients	Control subjects	ORa (95% CI)	p value	ORb (95% CI)	p value
**Age strata**							
60-79 yrs	51	17 (33.3)	56 (54.9)	0.36 (0.17-0.78)	0.010	0.32 (0.14-0.74)^d^	0.007
80 yrs or more	37	7 (45.9)	48 (64.9)	0.30 (0.10-0.86)	0.026	0.29 (0.09-0.91)^e^	0.034

**Risk strata^c^**							
Stratum 1	29	11 (37.9)	36 (62.1)	0.31 (0.11-0.89)	0.029	0.12 (0.03-0.53)^f^	0.005
Stratum 2	41	18 (43.9)	53 (64.6)	0.32 (0.13-0.79)	0.014	0.29 (0.11-0.79)^g^	0.015
Stratum 3	18	5 (27.8)	15 (41.7)	0.48 (0.12-1.91)	0.294	0.40 (0.09-1.89)^h^	0.249

NOTE: OR, odds ratio of vaccination versus no vaccination in cases and controls; CI, confidence interval.

^a^Mantel-Haenszel matched Odds Ratio.

^b^Matched, adjusted Odds Ratio by conditional logistic regression model.

^c ^Among the 29 case patients in risk stratum 1 (possible immunocompromised subjects), main underlying condition for matching was: immunodeficiency in 1 case, cancer in 11 cases (4 genitourinary, 3 haematological, 2 lung, 1 digestive, 1 breast), chronic renal disease in 12 cases (10 chronic renal failure, 1 nephrotic syndrome, 1 dialysis), and corticosteroid therapy in 5 cases. Among the 41 patients in risk stratum 2 (high-risk immunocompetent subjects), main underlying condition for matching was chronic lung disease in 24 cases, chronic liver disease in 3 cases, chronic heart disease in 8 cases and diabetes mellitus in 6 cases. Among the 18 patients in risk stratum 3 (non high-risk immunocompetent subjects), 6 were smokers, and they were matched to their controls according to this condition.

^d ^Adjusted for age, chronic heart disease, smoking and alcoholism.

^e ^Adjusted for age.

^f ^Adjusted for age, stroke and chronic heart disease.

^g ^Adjusted for age, diabetes mellitus, smoking and stroke.

^h ^Adjusted for age and smoking.

## Discussion

The effectiveness of PPV is controversial. Despite numerous studies, contradictory results have been reported and several meta-analyses have been inconclusive to date. Excluding earlier trials in younger adults,[21,22] prospective RCTs have failed to demonstrate a significant protective effect of the vaccination. However, several case-control and cohort studies have reported a considerable protective effect (ranging between 40-80%) in preventing IPD in different populations [23-28].

In general, meta-analyses of RCTs concluded that PPV did not prove any significant protective effect whereas those meta-analyses which also included observational studies in their analysis concluded that PPV could be considerably effective in preventing IPD (although its efficacy could be low or null among immunocompromised, high-risk and older adults) [7-17].

In the present study, a significant adjusted vaccine effectiveness of 72% (46-85) against overall IPD has been found. The effect of the vaccine in preventing vaccine-type IPD was greater, with an estimated effectiveness of 77% (40-92).

In our study, vaccination was significantly effective as for people 60-79 years as well as for older people 80 years or more. In addition, vaccination appears significantly effective for high-risk people and subjects with possible immunocompromise. Our findings fit with a prior case control study that showed an effectiveness of 66% against all bacteraemic pneumococcal pneumonia and 76% against vaccine-type infections[19].

Our results differ with those reported in the most recent meta-analysis by Huss et al, who concluded that PPV would be not efficacious against either IPD or pneumonia[17]. However, in the present authors opinion, with regard to IPD this conclusion exceeds the scope of the evidence from the meta-analysis, given that pneumococcal bacteraemia was a rare event in the included RCTs (with only 44 cases of pneumococcal bacteraemia from 6 studies including 32 770 participants) and confidence intervals (CI) of vaccine efficacy were extremely wide[17].

In contrast, our findings fit with those reported in the latest Cochrane systematic review,[16] which included 10 RCTs involving 35,483 participants assessing this outcome, showing a vaccine efficacy of approximately 74% (95% CI: 56% to 85%) against all IPD. In the mentioned Cochrane review, the pooled vaccine effectiveness based on 7 observational studies reached 52% (95% CI: 59% to 63%), which is also in agreement with the result observed in the present study.

Our data is also in agreement with data found in a prior cohort study among elderly people in the same geographical area, which pointed to an effectiveness of 40% against overall IPD[28]. Our result also accords with vaccine effectiveness against IPD reported in the case-control studies of Shapiro and Clemens (67%),[23] Sims et al (70%),[24] Farr et al (81%)[25] and Dominguez et al (72%)[26].

In the present study, stratified analyses according to age subgroups showed that vaccination was significantly effective for all people 60 years or older. We did not found evidence that vaccination effectiveness decreases with increasing age considering that in our study vaccine effectiveness was 68% (26-86) in people 60-79 years and it reached 71% (9-91) in people 80 years or more.

According to risk strata of the patients, in our study the pneumococcal vaccination was significantly effective in risk strata 1 (immunocompromised subjects) and 2 (immunocompetent subjects with any high-risk condition). Vaccination did not emerge significantly effective among the subgroup of patients in risk strata 3 (immunocompetent subjects without high-risk conditions); however, in order to interpret this result, it must be noted that our study was clearly underpowered to detect a possible benefit of vaccination in this subgroup considering the low vaccine coverage observed among these patients (27.8%) and the small number of case-control sets that contributed to this analysis (only 18 sets). Surprisingly, we did not find lower vaccine effectiveness among immunocompromised people in risk stratum 1. However, in order to interpret this result, it must be noted that this stratum included patients with possible (but not confirmed) immunocompromise and a few patients assigned to this risk stratum had conditions associated with severe immunocompromise (see footnote in table 5).

A limitation of case-control studies is their observational nature, which can lead to bias and confounding. We took care to avoid bias in selection of controls, by using rigorous methods to select control subjects and matching cases to controls as closely as possible according to the main risk factor and further adjusting for underlying diseases in the multivariable regression models. It must be noted that, in our study, case patients had a significant lower coverage of influenza vaccination than control subjects, and this could have contributed to a higher rate of pneumococcal infection among case subjects. Thus, considering influenza as a possible confounder, we made stratified analysis on vaccine effectiveness within influenza and noninfluenza periods and adjusting for influenza vaccine status. Nevertheless, a residual confounding on the estimates of vaccine effectiveness can not be completely excluded, perhaps because of undiagnosed illnesses, differences in functional status of the patients or other epidemiological factors not considered in this study.

The study's major strengths were that it was population-based, and the study population (people 60 years or older with and without chronic illness) was representative and large enough to evaluate the most specific outcome (IPD due to vaccine serotypes) related with pneumococcal vaccination in adults. In addition, vaccine effectiveness was estimated adjusting for important covariables such as age, underlying conditions and influenza vaccine status. Thus, although it was not a RCT, it provides an adequate basis for assessing the potential benefit of the 23-valent PPV in preventing IPD among people 60 years or older, with and without high-risk conditions.

## Conclusions

Some studies have reported that the PPV is cost-effective for preventing IPD among people 65 years or older in the USA and western European countries,[29,30] but reluctance to use this vaccine exists and current uptakes are still low in many countries. The benefit of vaccination for the general elderly and high-risk individuals, as it has been reported in this study, supports the widespread use of the vaccine. However, it must not be forgotten that the vaccine provides incomplete protection, so the development of more effective vaccination strategies for adults are still necessary. In this way, efforts to develop new conjugated vaccines with high-serotype coverage for adults, and investigations into new technology protein-based vaccines with potential serotype-independent protection against pneumococcal infections are greatly needed [31,32].

## Competing interests

The authors declare that they have no competing interests.

## Authors' contributions

AVC designed the study; IH, ES and CMF conducted data collection; EPIVAC Study Group collaborated in the data collection, AVC and OO supervised the data collection; AVC wrote the draft of the manuscript; TRB did the statistical analysis; all authors read and approved the final manuscript.

## Pre-publication history

The pre-publication history for this paper can be accessed here:

http://www.biomedcentral.com/1471-2334/10/73/prepub
